# Brain morphometric abnormalities and their associations with affective symptoms in males with methamphetamine use disorder during abstinence

**DOI:** 10.3389/fpsyt.2022.1003889

**Published:** 2022-10-10

**Authors:** Xinyue Hu, Ping Jiang, Yingxue Gao, Jiayu Sun, Xiaobo Zhou, Lianqing Zhang, Hui Qiu, Hailong Li, Lingxiao Cao, Jing Liu, Qiyong Gong, Xiaoqi Huang

**Affiliations:** ^1^Functional and Molecular Imaging Key Laboratory of Sichuan Province, Department of Radiology, Huaxi Magnetic Resonance Research Center (HMRRC), West China Hospital, Sichuan University, Chengdu, China; ^2^Psychoradiology Research Unit of the Chinese Academy of Medical Sciences, West China Hospital of Sichuan University, Chengdu, China; ^3^West China Medical Publishers, West China Hospital of Sichuan University, Chengdu, China; ^4^Department of Radiology, West China Hospital of Sichuan University, Chengdu, China; ^5^Department of Psychosomatics, Academy of Medical Sciences and Sichuan Provincial People's Hospital, Chengdu, China

**Keywords:** methamphetamine use disorder, abstinence, cortical morphometry, subcortical volume, affective symptoms

## Abstract

**Background:**

Methamphetamine (METH) use induces neurotoxic effects in brain structures and affective symptoms that persist during abstinence. However, the brain morphometry of individuals with METH use disorder (MUD) remains unclear, as well as their associations with affective symptoms during abstinence.

**Methods:**

Forty-eight abstinent males with MUD and 66 age-, sex-, and education-matched healthy controls (HCs) underwent high-resolution T1-weighted magnetic resonance imaging. Cortical thickness, surface area, volume, local gyrification index (LGI), and subcortical volume were obtained with FreeSurfer software. Brain morphometry differences between groups and their associations with affective symptoms and drug abuse history within the males with MUD were examined, with intracranial volume, age, and years of education as covariates.

**Results:**

Compared with the HCs, the individuals with MUD showed a significantly higher LGI in the right cuneus gyrus, left lingual gyrus, bilateral supramarginal gyrus, right inferior parietal gyrus (IPG), and right dorsal anterior cingulate cortex (clusterwise *p* < 0.05, Monte Carlo-corrected), as well as a smaller volume of the left nucleus accumbens (NAcc) (*p* < 0.05, FDR-corrected). However, there were no significant group differences in cortical thickness, area or volume. In addition, the LGI in the right IPG was positively associatedwith the severity of depression and anxiety symptoms in MUDs (*p* < 0.05, FDR-corrected).

**Conclusion:**

Brain morphometric abnormalities in abstinent males with MUD were characterized by hypergyrification across multiple mid-posterior brain regions anda smaller volume of the left NAcc.Gyrification of the right IPG may be a potential neural substrate underlying the affective symptoms experienced by MUDs during abstinence.

## Introduction

Methamphetamine (METH) is an amphetamine-type stimulant (ATS) that has high dopamine (DA)-related neurotoxicity in the mesocorticolimbic system (1). Chronic METH use can result in severe behavioral, cognitive, and memory impairments and symptoms of psychosis (2). Affective symptoms, including anxiety and depression, are frequently observed in individuals with methamphetamine use disorder (MUD) during abstinence (3). Affective symptoms have been shown to exacerbate relapse of METH use, craving and prolong treatment, especially in individuals with MUD early in the abstinence period (3–5). However, no medication-based interventions are available to effectively treat MUD and its related affective symptoms (2). A few brain structure studies have explored the relationships between brain structures and affective symptoms in individuals with MUD. For example, one study reported that decreased CT in the inferior temporal, orbitofrontal, and inferior frontal gyri was associated with dysfunction of affective regulation in individuals with MUD (6). However, the neuroanatomical basis of METH-related affective symptoms remains poorly understood, although it is critical for the development of treatment strategies for this population and thus for improving patient care and preventing relapse.

Converging evidence from pathological and neuroimaging studies in human drug users and animal models has suggested that chronic METH use can contribute to structural brain abnormalities (7–10). Structural brain abnormalities in individuals with MUD have been widely reported in the mesocorticolimbic system, including the regions of the prefrontal cortex (PFC), anterior cingulate cortex (ACC), hippocampus, amygdala, and nucleus accumbens (NAcc) (9, 11). Compared with healthy controls (HCs), abstinent MUDs displayed smaller gray matter volume (GMV) in the right lateral occipital cortex and decreased cortical thickness (CT) in the bilateral superior frontal cortex (12). Another study reported larger GMV in the striatum in MUDs than in HCs (13). However, there have not been sufficiently powered studies to comprehensively investigate sex differences in specific brain morphometric features in MUDs who only used METH (excluding multiple drug use).

To date, most previous studies of brain morphometric alterations in MUDs have focused on volumetric measures using voxel-based morphometry (VBM). However, a more comprehensive examination of abnormalities in brain morphometry in MUD individuals using surface-based morphometry (SBM), including CT, surface area (SA), cortical volume (CV), and local gyrification index (LGI), may advance our understanding of the effects of METH on the brain. These measures are influenced by distinct evolutionary, neurodevelopmental, and genetic factors in different ways (14–16). CT primarily reflects the number of neurons within a cortical column, starts to decreases from the age of 2–4 years and continues throughout the lifespan (17–19). SA is related to the number of cortical mini-columns, expands until about the age of 12 years, remains relatively stable and then shrinks with age (20). CV reflects the properties of both CT and SA, which is more closely related to SA rather than CT and follows a non-monotonic and non-linear developmental trajectory (17). The LGI reflects the degree of cortical gyrification, progressively increases in the first 2 years of life and then decreases throughout the lifespan (21–23).

Based on this background, our study aimed to investigate abnormal brain morphometry using multiple brain morphometric features, including CT, SA, CV, and the LGI, as well as subcortical volume in abstinent males with a history of METH abuse alone compared with age-, sex-, and education-matched HCs. The MUD participants recruited in the current study had a very low level of smoking (<1 cigarette per day), thereby eliminating the confounding effects of polysubstance abuse and assisting in the determination of the “pure” effect of METH on brain morphometry. In addition, we explored the relationships between abnormal brain morphometry and affective symptoms in MUDs. We hypothesized that the brain morphometric alterations would be observed in the PFC, ACC, and NAcc which are the key brain regions in the mesocorticolimbic system and that some of the abnormalities would be related to affective symptoms in the MUDs during abstinence.

## Methods

### Participants

This study was approved by the Research Ethics Committee of West China Hospital, Sichuan University, and fully informed written consent was obtained from all participants. Participation was entirely voluntary. Forty-eight male MUD participants during abstinence (mean age: 28.77 years, SD: 7.66 years) and 66 age-, sex-, and education-matched HCs (mean age: 30.85 years, SD: 7.97 years) were recruited for the study. All participants were native Han Chinese and right-handed. MUDs were recruited from Ziyang, a compulsory isolation and rehabilitation center in Sichuan Province, China. The MUDs eligible for our study were at least 16 years old and able to understand and complete the measurements. METH abuse was diagnosed based on the criteria of the Diagnostic and Statistical Manual of Mental Disorders, 4th edition (DSM-IV). The exclusion criteria were (1) a history of use of or dependence on any psychoactive substances other than METH or nicotine; (2) a history of mental disorders before METH abuse; (3) major systemic diseases or neurological disorders, including HIV and diabetes; or (4) any contraindications to magnetic resonance imaging (MRI).

HCs were recruited from the local community through posters and flyers distributed at West China Hospital of Sichuan University and through internet advertisements. The same exclusion criteria were applied to HCs except that individuals with any history of drug use were excluded.

### Clinical assessment battery

METH abuse history and affective symptom assessments were recorded through a detailed interview by two experienced psychiatrists (XH and XZ) before the MRI scans.

In the MUD participants, the 17-item Hamilton Depression Scale (HAMD-17) and the 14-item Hamilton Anxiety Scale (HAMA-14) were used to evaluate the severity of depressive and anxiety symptoms, respectively. In both scales, a higher total score indicated more severe anxiety or depressive symptoms.

### MRI data acquisition

High-resolution 3D T1-weighted images were acquired using a 3-T MR scanner (Trio Tim, Siemens Healthineers, Erlangen, Germany) with a 12-channel phase-array head coil. Foam padding and soft earplugs were used to reduce head motion and scanner noise, respectively. A magnetization-prepared rapid gradient-echo (MPRAGE) sequence was used with the following parameters: repetition time (TR) = 1,900 ms, echo time (TE) = 2.26 ms, flip angle = 9°, matrix = 256 × 256, field of view (FOV) = 256 × 256 mm^2^, number of axial slices = 176, and slice thickness = 1.0 mm. All images were visually inspected by an experienced radiologist (J. Sun) during imaging, and those with head movement artifacts were immediately rescanned.

### MRI data pre-processing

The T1-weighted images were analyzed using the mainstream recon-all process of FreeSurfer software (version 6.0) (http://surfer.nmr.mgh.harvard.edu/). The image processing pipeline included visual inspection of data for motion artifacts, removal of non-brain tissue, transformation to Talairach space, segmentation of subcortical gray/white matter (GM/WM), intensity normalization, tessellation of the GM/WM boundary, automated topology correction, and surface deformation (24–26). The cortical surface then underwent inflation, registration to a spherical atlas, and automatic identification of gyral and sulcal regions.

CT was defined as the shortest straight-line distance between the pial surface and the GM/WM boundary (27). SA was obtained by assigning an area to each vertex equal to the average of its surrounding triangles (28). CV was obtained by calculating the amount of GMV within the pial surface and the GM/WM boundary (17). The LGI was obtained by quantifying local cortical folding by calculating the ratio of the amount of cortex buried within the sulcal folds relative to the amount of cortex on the outer visible cortical hull in a 25-mm spherical region (16). Vertex-level CT, SA, CV, and LGI in each subject were projected onto a targeted and normalized surface (“fsaverage”).

The volumes of subcortical nuclei, including the bilateral thalamus, caudate, putamen, pallidum, hippocampus, amygdala, and NAcc (**Figure 2A**), and the intracranial volume (ICV) were extracted from FreeSurfer's segmentation stream.

### Statistical analysis

#### Group comparison of brain morphometry

We investigated group differences in demographic characteristics (age, years of education, and ICV) between the MUD and HC groups using an independent two-sample *t*-test. Statistical analyses of cortical morphometry were conducted with the FreeSurfer Query, Design, Estimate, Contrast (Qdec) program (http://www.freesurfer.net/fswiki/Qdec). First, the CT, SA, and CV maps were spatially smoothed with a full-width at half-maximum Gaussian kernel of 10 mm (the LGI map was not smoothed due to its intrinsic smoothness). Second, we used a general linear model (GLM) to test for group differences in CT, SA, CV, and the LGI in a vertex-by-vertex manner, with diagnosis as a fixed factor and age, years of education, and ICV as covariates. A Monte Carlo simulation was used to correct for multiple hypothesis testing, with 10,000 iterations, cluster-forming *p* < 0.01 and clusterwise probability (CWP) < 0.05.

Analyses of group differences in the volumes of subcortical nuclei were tested using a multivariate analysis of covariance (MANCOVA), with age, years of education, and ICV as covariates. We used partial eta squared (η^2^) to evaluate effect size (0.01 indicates a small effect size, 0.06 indicates a medium effect size and 0.14 indicates a large effect size). A false discovery rate (FDR) correction was applied to correct for multiple comparisons in the subcortical nuclei analyses.

#### Correlations with affective symptoms

To examine the relationship between brain regions with significant group differences and clinical features (abstinent days, usage duration, mean dose (g/time), mean dose (g/day), onset age of METH use, HAMD score, and HAMA score), mean measurements within each region were extracted, and partial rank correlation analyses were performed due to their non-normal distribution after controlling for age, years of education, and ICV. In addition, we used Spearman correlation analyses to examine correlations between METH abuse history [abstinent days, usage duration, mean dose (g/time), mean dose (g/day), and onset age of METH use] and affective symptoms (HAMD and HAMA scores). An FDR correction was applied to correct for multiple comparisons in the correlation analyses.

## Result

### Demographic and clinical characteristics

The demographic and clinical characteristics of the abstinent males with MUD and HCs are presented in Table 1. The MUDs and HCs did not differ significantly in terms of age, years of education, or ICV.

**Table 1:** Demographic and clinical data of the male abstinent MAs and HCs.

	**MA (*N* = 48)**	**HC (*N* = 66)**	***t*** **-value**	***p-*** **value**
**Demographics**				
Age (years)	28.77 ± 7.66	30.85 ± 7.97	1.464	0.146
Education (years)	8.27 ± 3.68	8.66 ± 3.21	0.732	0.466
**Affective symptoms**				
HAMA score	3.56 ± 5.09			
HAMD score	4.90 ± 5.25			
**METH use**				
Abstinent periods (days)	114.56 ± 116.20	–		
Duration of use (months)^a^	47.74 ± 39.62	–		
Mean dose (g/time)	0.39 ± 0.30	–		
Mean dose (g/day)	0.94 ± 1.49			
Age of first use (years)^a^	24.66 ± 8.20	–		

a N = 47. Data are presented as the means ± standard deviation. MA, methamphetamine abuser; HC, healthy control; METH: methamphetamine; HAMD: Hamilton depression scale; HAMA: Hamilton anxiety scale; g, gram.

### Group differences in brain morphometry

**Figure 1:**
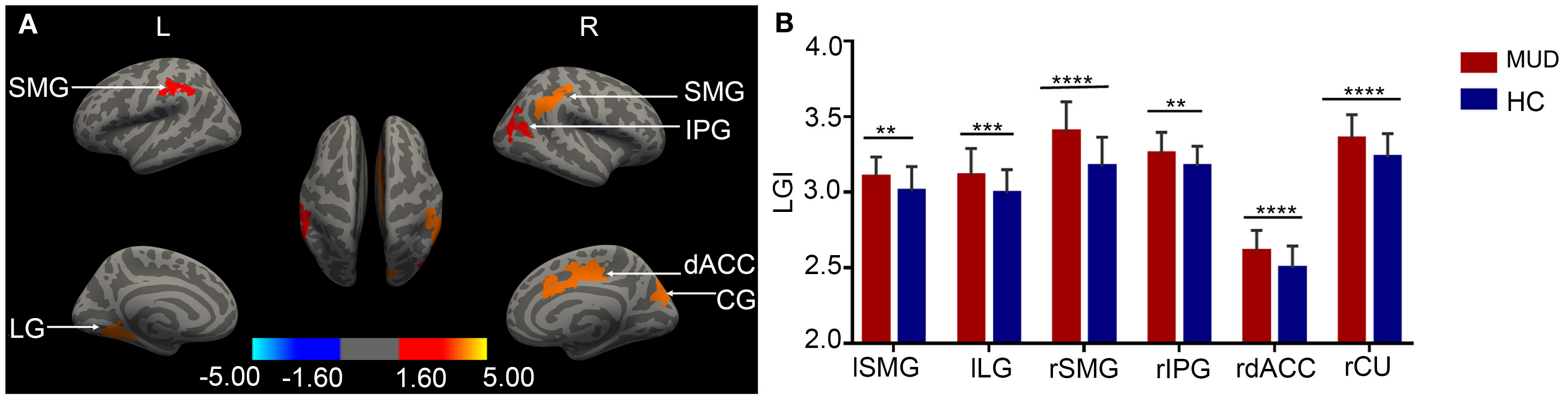
**(A)** Cortical clusters with significantly higher LGI values in MUD participants than HCs (CWP < 0.05, Monte Carlo-corrected). None of the significant clusters for cortical thickness, surface area or volume survived the correction. The color bar for p values is on a logarithmic scale (log_10_) with a range of 1.6–5. **(B)** The group differences in LGI values are illustrated by representative bar plots. MUD, methamphetamine use disorder; HC, healthy control; LGI, local gyrification index; L/l, left; R/r, right; SMG, supramarginal gyrus; LG, lingual gyrus; IPG, inferior parietal gyrus; dACC, dorsal anterior cingulate cortex; CU, cuneus. CWP, clusterwise probability. **p < 0.01; ***p < 0.001, ****p < 0.0001.

Compared with HCs, the MUDs showed higher LGI values mainly in the bilateral supramarginal gyrus (SMG), left lingual gyrus (LG), right inferior parietal gyrus (IPG), right cuneus (CU) and right dorsal anterior cingulate cortex (dACC) (CWP < 0.05, Monte Carlo-corrected, Table 2 and Figures 1A,B). Notably, these deficits were located primarily in the mid-posterior cortex. However, there were no significant differences in CT, SA, or CV between the MUDs and HCs.

**Table 2:** Significant group differences in brain morphometry between the abstinent males with methamphetamine use disorder and HCs.

**Significant group differences in LGI with age, education years, and ICV as covariates**							
**Brian** **morphometric**	**Anatomical** **location**	**Direction**	**MNI coordinates (peak vertex)**			**Size** **(mm^2^)**	**CWP**
			**x**	**y**	**z**		
LGI	Left supramarginal gyrus	MUD > HC	−51.4	−27.5	32.6	975.65	0.0012^**^
LGI	Left lingual gyrus	MUD > HC	−24.1	−51.0	−1.9	1,363.90	<0.001^***^
LGI	Right supramarginal gyrus	MUD > HC	54.1	36.7	40.6	1,694.14	<0.001^***^
LGI	Right inferior parietal gyrus	MUD > HC	47.1	−69.8	13.9	907.08	0.0012^**^
LGI	Right cuneus gyrus	MUD > HC	11.8	−74.9	27.3	1,044.67	<0.001^***^
LGI	Right dorsal anterior cingulate cortex	MUD > HC	13.6	−17.0	36.0	1,361.74	<0.001^***^
**Significant group differences in volumes (mm** ^3^ **) of subcortical nucleus with age, education years, and ICV as covariates**							
**Volume**	**Volume in MUD (mm** ^3^ **)**	**Volume in HC (mm** ^3^ **)**	***F*** **-value**	η^2^	**FDR-corrected** ***p-*****value**		
Left NAcc	446.47 ± 89.82	517.38 ± 53.25	8.336	0.071	0.035^*^		

There were no significant differences in CT, SA, or CV between the MUDs and HCs. MUD, methamphetamine use disorder; HC, healthy control; MNI, Montreal neurological institute; LGI, local gyrification index; CWP, cluster-wise probability; NAcc, nucleus accumbens; CT, cortical thickness; SA, surface area; CV, cortical volume; FDR, false discovery rate.

* p < 0.05,

** p < 0.01;

*** p < 0.001.

**Figure 2:**
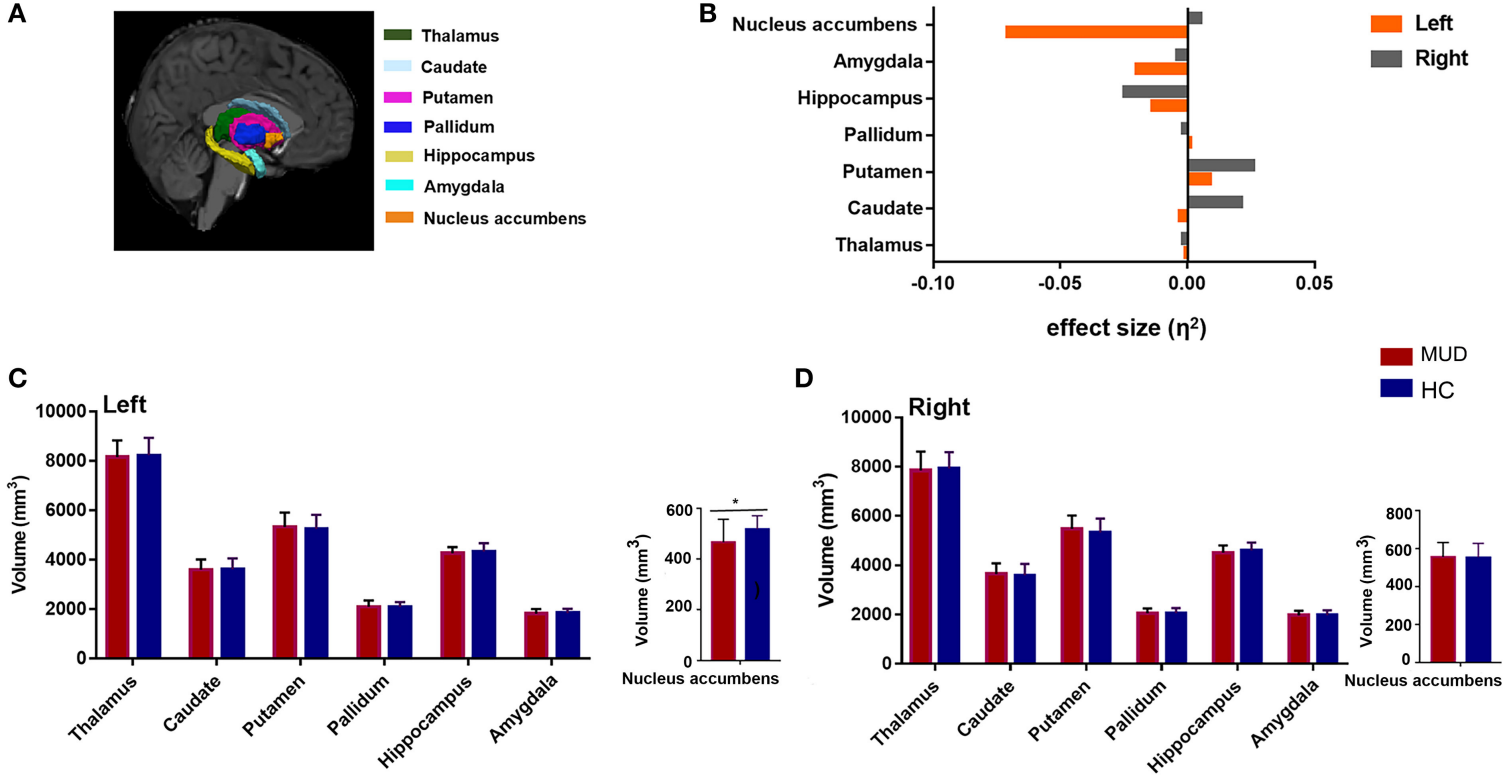
**(A)** An example of subcortical nucleus segmentation by FreeSurfer (version 6.0) in a healthy subject (right subcortical nucleus is shown). **(B)** Effect sizes for differences in left (gray) and right (orange) subcortical nuclei between the MUDs and HCs. **(C,D)** Bar plots of volumes (mm^3^) of the bilateral subcortical nucleus in the MUD participants and HCs after controlling for age, years of education and ICV. *Indicates significance after FDR correction. MUD, methamphetamine use disorder; HCs, healthy control; ICV, intracranial volume; FDR, false discovery rate; L, left; R, right.

Compared with HCs, the MUDs also showed a significantly smaller volume of the left NAcc (*F* = 8.366, FDR-corrected *p* = 0.035, *η*^2^ = 0.071; Table 2 and Figure 2). Significant differences in other nuclei were not observed between the two groups (Figures 2B–D).

### Correlations with affective symptoms

**Figure 3:**
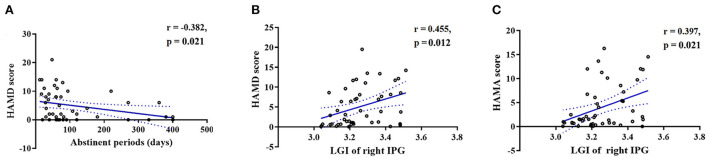
Scatterplots showing that the duration of abstinence was significantly negatively correlated with **(A)** HAMD scores in the MUDs. Scatterplots showing that the LGI in the right IPG was significantly positively correlated with the **(B)** HAMD and **(C)** HAMA scores in the MUDs. All of the abovementioned correlations, with the exception of those between the duration of abstinence and HAMA scores, remained significant after FDR correction. MUD, methamphetamine use disorder; HAMD, Hamilton depression scale; HAMA, Hamilton anxiety scale; LGI, local gyrification index; IPG, inferior parietal gyrus; FDR, false discovery rate.

HAMD scores were negatively correlated with the duration of abstinence (*r* = −0.382, FDR-corrected *p* = 0.021, Figure 3A), whereas both HAMD scores (*r* = 0.455, FDR-corrected *p* = 0.012, Figure 3B) and HAMA scores (*r* = 0.397, FDR-corrected *p* = 0.021, Figure 3C) were positively correlated with the LGI in the right IPG.

## Discussion

In this study, we used multiple cortical and subcortical measures to investigate a comprehensive profile of morphometric abnormalities of the brain anatomy in the MUDs with several findings. First, comparing to HCs, we observed increased LGI in the bilateral SMG, left LG, right IPG, right CU, and right dACC. *Second*, we loated reduced volume of the left NAcc in the MUDs relative to HCs. *Second*, we found the LGI in the right IPG positively correlated with the severity of anxiety and depressed symptoms in the MUDs. Overall, these findings suggest that the gyrification of mid-posterior cortex are disrupted in the MUDs and that right IPG may serves as a neural substrate underlying affective symptoms in the MUDs.

### Brain morphometric abnormalities in the MUDs

We found significant group differences in the LGI analyses; specifically, the MUDs showed significant hypergyrification in the right CU, left LG, bilateral SMG, right IPG, and right dACC regions compared with HCs. The LGI is a 3D metric used to quantify the degree of cortical gyrification, which reflects cortical complexity (16). A cortex with extensive gyrification has a high LGI, whereas a cortex with limited gyrification has a low LGI (16). Several factors influence cortical gyrification, including neuronal proliferation and migration, axonal connectivity, and mechanical constraints (29). Previous neuroimaging studies have reported that patterns of cortical gyrification are crucial for shaping different brain functions (30–32). Therefore, our observation of aberrant LGI values across the mid-posterior cortex may be related to corresponding cognitive and behavioral impairments.

Previous neuroimaging evidence has consistently demonstrated that METH abuse contributes to impairments in several neuropsychological functions, including executive functions, such as visual memory, verbal processing and cognitive control/response inhibition, and social cognition, such as empathy, communication, and facial-emotion recognition (33). In our study, the brain regions exhibiting hypergyrification in the MUDs have also been reported to play critical roles in executive function and social cognition. For example, the LG and CU are important for visual processing, and the IPG is associated with visual, auditory, and sensorimotor integration (34–36). The right SMG plays a key role in controlling empathy toward other people (37), and the left SMG is important in language perception and processing (38). In addition, the dACC plays a critical role in executive function, especially in reward-based decision making (39). Consistent with our morphological observations, dysfunctions in the SMG and ACC during tasks that require executive function, and social cognition in early abstinent MUDs have also been reported (40). Therefore, hypergyrification in the mid-posterior brain regions reported here may be the neural-structural basis for impairments in executive function and cognition in MUDs. Moreover, as the neuronal development of gyrification is completed within the first 2 years of life, we boldly suggested that those identified regions with LGI alterations in the MUDs might be serve as vulnerability factors for METH abuse. Meanwhile, the relationship between gyrification in the dACC and executive function is still unclear, might be explored in future studies.

Our findings also showed a smaller volume of the left NAcc in the MUD s relative to HCs; similar findings have been reported by other neuroimaging studies with MUDs (11, 41). This observation supports our hypothesis that brain regions within the DA reward circuit are the main targets of the METH-induced neurotoxic effects. The NAcc is considered the main part of the ventral striatum (33), which receives rich dopaminergic input from the VTA and is an important component of the “reward circuit” of the brain, with functions related to the mediation of natural and drug rewards (42, 43). METH use leads to acute reward and reinforcement primarily *via* the release of massive amounts of DA in the reward circuit, including in the NAcc (33). Moreover, prolonged METH use results in neurotoxic effects, including declines in DA receptors and transporters as well as neurite degeneration (44–46). Since GMV correlated with DA receptor ligand binding (47), and reduced DA transporter density and DA receptors in the striatum had been detected in methamphetamine abusers in previous study (48), we postulated that volumetric alterations in the NAcc in MUDs probably reflect neuronal biochemical changes induced by METH.

Interestingly, among all four cortical parameters (CT, SA, CV, and the LGI), only the LGI showed significant group differences, suggesting that the LGI may be a more sensitive biomarker for abnormalities in cortical morphometry in MUDs during abstinence. Therefore, understanding the complex changes in cortical gyrification may contribute to current understanding of the effects of METH abuse on brain structure. However, our observations are inconsistent with some previous brain morphometric studies on METH abuse (12, 49). One study reported reduced CT in posterior cingulate gyrus in MUDs with including histories of marijuana abuse or dependence, compared with HCs (50). Another study reported increased CT in the parietal cortex in MUDs with a long-term (14–25 months) compulsory abstinence relative to HCs (51). Nie et al. (12) reported increased CT in the bilateral superior frontal gyri in MUDs compared to those in HCs. We hypothesis the discrepancies on CT alterations in our study with previous ones maybe due to the demographic and clinical characteristic difference (such as polysubstance abuse, duration of abstinence, and different sample sizes) among studies. And our study bears the advantage of single drug abuse.

### Associations between brain morphometric abnormalities and affective symptoms in MUDs

Our exploratory analysis found that the LGI in the right IPG was positively associated with HAMD and HAMA scores in the MUDs. Previous studies have reported that aberrant changes in the LGI are related to emotion regulation in several psychiatric disorders, including major depression disorder, generalized anxiety disorder, and bipolar disorder (31, 52, 53).

One of the most influential perspectives posits that cortical gyrification is, to a large extent, induced by axonal tension between local brain regions that pull on the nearby cortex (21), influencing the formation of functional connectivity between these regions (54, 55). Therefore, a higher LGI may reflect long-range hypoconnectivity between brain regions (30); our observation of a higher LGI in the right IPG may imply hypoconnectivity between the right IPG and other brain regions in MUDs. The IPG is an important node in the default mode network (DMN) that is critically involved in the rumination process, and its dysfunction is a well-documented risk factor for the onset of depressive and anxiety symptoms (56). Therefore, aberrant gyrification in the IPG as described by this study may induce dysfunction of the DMN that is associated with depressive and anxiety symptoms, which is in line with our previous finding that intranetwork functional connectivity determines the severity of affective symptoms in MUDs (57). This result also suggests that the abnormal LGI in the right IPG in our MUDs may be a potential neural mechanism for METH use-induced affective symptoms.

The current study has several limitations. First, the cross-sectional design prevents the drawing of a causal relationship between brain morphometric abnormalities drug abuse as well as associated affective symptoms in MUDs. Longitudinal studies are encouraged to address these issues. Second, our sample was comprised of only male MUDs and thus cannot represent brain morphometric differences in female MUD participants. Since sex hormones may be an important factor in brain morphometric differences (41, 58), future work with female MUDs and investigations of sex differences are needed. Third, the MUDs were recruited from a compulsory isolation and rehabilitation center, which might limit the generalization of our findings. Future research with MUDs recruited from local communities is necessary to obtain generalizable results.

In summary, we found that the brains of abstinent males with MUD appeared to be characterized by hypergyrification across multiple mid-posterior brain regions involved in processing language, vision and emotion; the MUDs possessed significantly smaller left NAcc volumes, an area involved in reward processing. In addition, gyrification in the right IPG was positively associated with the severity of affective symptoms in MUDs, suggesting that it may be a potential neural mechanism underlying the affective symptoms experienced by MUDs during abstinence.

## Data availability statement

The raw data supporting the conclusions of this article will be made available by the authors, without undue reservation.

## Ethics statement

The studies involving human participants were reviewed and approved by the Research Ethics Committee of West China Hospital, Sichuan University. The patients/participants provided their written informed consent to participate in this study.

## Author contributions

XHu, PJ, XHua, and QG formulated the research questions. XHua and QG designed the study. JS, XZ, and HL acquired the data. XHu, LZ, YG, LC, and HQ analyzed the data. XHu, PJ, YG, XHua, and QG interpreted the data and wrote or revised the article. All authors approved the final version for publication.

## Funding

This study was supported by grants from the 1.3.5 Project for Disciplines of Excellence, West China Hospital, Sichuan University (Grant No. ZYJC21041), the Clinical and Translational Research Fund of Chinese Academy of Medical Sciences (Grant No. 2021-I2M-C&T-B-097), and the Natural Science Foundation of Sichuan Province (Grant Nos. 2022NSFSC0052 and 2022NSFSC1454).

## Conflict of interest

The authors declare that the research was conducted in the absence of any commercial or financial relationships that could be construed as a potential conflict of interest.

## Publisher's note

All claims expressed in this article are solely those of the authors and do not necessarily represent those of their affiliated organizations, or those of the publisher, the editors and the reviewers. Any product that may be evaluated in this article, or claim that may be made by its manufacturer, is not guaranteed or endorsed by the publisher.
